# Diethyl [hydr­oxy(phen­yl)meth­yl]phospho­nate

**DOI:** 10.1107/S1600536808018424

**Published:** 2008-06-21

**Authors:** Li-Tao An, Gui-Xia Gong, Xing Liu, Min Xia, Jian-Feng Zhou

**Affiliations:** a Jiangsu Key Laboratory for the Chemistry of Low-dimensional Materials, Department of Chemistry, Huaiyin Teachers College, Huaian 223300, Jiangsu Province, People’s Republic of China; b Key Laboratory of Organic Synthesis of Jiangsu Province, College of Chemistry and Chemical Engineering, Suzhou University, Suzhou 215123, People’s Republic of China; c Department of Chemistry and Biology, Yulin Normal University, Yulin 537000, People’s Republic of China

## Abstract

Mol­ecules of the title compound, C_11_H_17_O_4_P, are linked into chiral helical chains along the crystallographic *b* axis *via* O—H⋯O hydrogen bonds between the hydr­oxy group and an O atom of the phospho­nate group. One ethyl group is disordered over two positions; the site occupancy factors are *ca* 0.7 and 0.3.

## Related literature

For related literature, see: Fang *et al.* (2006*a* ▶,*b* ▶,*c* ▶, 2007 ▶); Kaboudin (2000 ▶); Maier & Diel (1994 ▶); Stowasser *et al.* (1992 ▶).
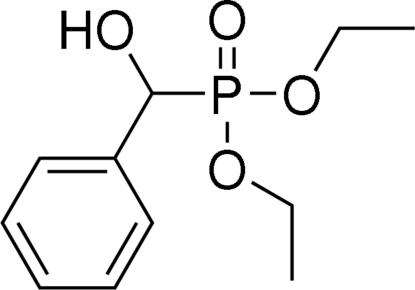


## Experimental

### 

#### Crystal data

C_11_H_17_O_4_P
*M* *_r_* = 244.22
Monoclinic, 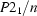

*a* = 9.2361 (6) Å
*b* = 8.0719 (5) Å
*c* = 17.4599 (13) Å
β = 95.096 (5)°
*V* = 1296.54 (15) Å^3^
*Z* = 4
Mo *K*α radiation
μ = 0.21 mm^−1^
*T* = 296 (2) K
0.30 × 0.30 × 0.20 mm

#### Data collection

Rigaku Mercury diffractometer
Absorption correction: multi-scan (Jacobson, 1998 ▶) *T*_min_ = 0.940, *T*_max_ = 0.959
10679 measured reflections
2345 independent reflections
1723 reflections with *I* > 2σ(*I*)
*R*_int_ = 0.030

#### Refinement

*R*[*F* ^2^ > 2σ(*F* ^2^)] = 0.055
*wR*(*F* ^2^) = 0.174
*S* = 1.07
2345 reflections
168 parameters
48 restraints
H-atom parameters constrained
Δρ_max_ = 0.38 e Å^−3^
Δρ_min_ = −0.34 e Å^−3^

### 

Data collection: *CrystalClear* (Rigaku/MSC, 2001 ▶); cell refinement: *CrystalClear*; data reduction: *CrystalStructure* (Rigaku/MSC, 2004 ▶); program(s) used to solve structure: *SHELXS97* (Sheldrick, 2008 ▶); program(s) used to refine structure: *SHELXL97* (Sheldrick, 2008 ▶); molecular graphics: *ORTEP-3* (Farrugia, 1997 ▶); software used to prepare material for publication: *SHELXL97*.

## Supplementary Material

Crystal structure: contains datablocks global, I. DOI: 10.1107/S1600536808018424/rk2099sup1.cif

Structure factors: contains datablocks I. DOI: 10.1107/S1600536808018424/rk2099Isup2.hkl

Additional supplementary materials:  crystallographic information; 3D view; checkCIF report

## Figures and Tables

**Table 1:** Hydrogen-bond geometry (Å, °)

*D*—H⋯*A*	*D*—H	H⋯*A*	*D*⋯*A*	*D*—H⋯*A*
O1—H1⋯O2^i^	0.82	1.90	2.716 (3)	174

Symmetry code: (i) 
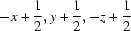
.

**Fig. 1.**
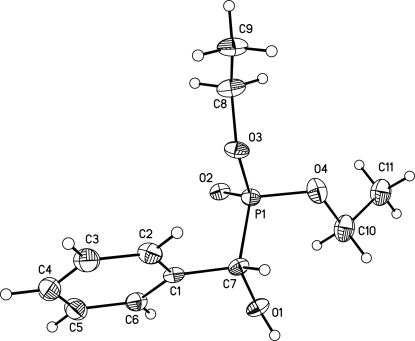
A molecular structure of I with the atom numbering scheme. Displacement ellipsoids are drawn at 30% probability level. Only major part of disoprdered moiety are presented. The H atoms are drawn as a small spheres of arbitrary radius.

**Fig. 2.**
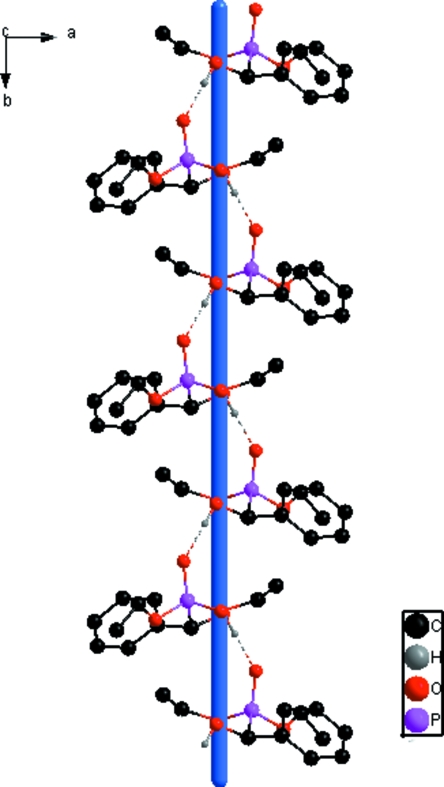
The chiral helical chain constructed by O—H···O H–bonds.

**Fig. 3.**
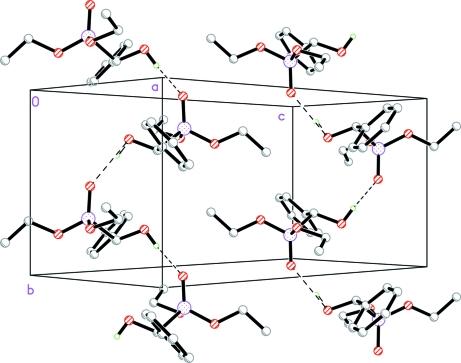
Part of the crystal structure of I, showing chiral helical chains aligned in an antiparallel fashion. The H atoms not involved in H–bonds are omitted for clarity.

## supplementary crystallographic information

### Comment

The α–hydroxyphosphonic esters and their derivatives have attracted considerable interest owing to their interesting biological activities, such as inhibition of inositol monophosphatase (Maier & Diel, 1994) and HIV protease (Stowasser *et al.*, 1992). These compounds are generally synthesized from aldehydes and phosphites *via* the base–catalyzed Pudovik reaction, as exemplified by diisopropyl (hydroxyphenylmethyl)phosphonate (Fang, *et al.*, 2006*a*), dimethyl [hydroxy(phenyl)methyl]phosphonate (Fang, *et al.*, 2006*b*), diphenyl (hydroxyphenylmethyl)phosphonate (Fang, *et al.*, 2006*c*) and diethyl hydroxy(4–methoxyphenyl)methylphosphonate (Fang, *et al.*, 2007). As an extension of these studies, we report herein on the structure of C_11_H_17_O_4_P, (I), (Fig. 1).

In I, the C10 and C11 atoms are disordered over two sets of sites with occupancies of 0.727 (7) and 0.273 (7). All bond distances and bond angles of I are normal and call for no further comment.

There exist a strong intermolecular H-bonding between O1—H1···O2^i^ (symmetry code: (i) 1/2-*x*, 1/2+*y*, 1/2-*z*) in I. Molecules of I are linked into chiral helical chains by this H–bonding, running parallel to the *b* axis (Fig. 2). But these chains are aligned in an antiparallel fashion to form inversion centers in the crystal, thus the whole structure is achiral (Fig.3).

### Experimental

All chemicals were obtained from commercial sources and used directly without further purification. Magnesium oxide (2 g) was added to a stirred mixture of diethyl phosphite (0.02 mol) and aldehyde (0.02 mol) at room temperature. After 2 h the mixture was washed by dichloromethane (50 ml) and dried with CaCl_2_; evaporation of the solvent gave the crude product. The products were crystallized from *n*–hexane (Kaboudin, 2000).

### Refinement

All H atoms were positioned geometrically and were allowed to ride on their parent atoms, with C—H distances of 0.93–0.97Å (0.82Å for O—H group) and *U*_iso_(H) values constrained to be 1.2 (1.5 for –OH and –CH_3_ group) times *U*_eq_ of the carrier atom.

### Crystal data

**Table:**

C_11_H_17_O_4_P	*Z* = 4
*M**_r_* = 244.22	*F*_000_ = 520
Monoclinic, *P*2_1_/*n*	*D*_x_ = 1.251 Mg m^−^^3^
Hall symbol: -P 2yn	Mo *K*α radiation λ = 0.71073 Å
*a* = 9.2361 (6) Å	θ = 2.3–25.2º
*b* = 8.0719 (5) Å	µ = 0.21 mm^−^^1^
*c* = 17.4599 (13) Å	*T* = 296 (2) K
β = 95.096 (5)º	Prism, colourless
*V* = 1296.54 (15) Å^3^	0.30 × 0.30 × 0.20 mm

### Data collection

**Table:**

Rigaku Mercury diffractometer	2345 independent reflections
Radiation source: Fine–focus sealed tube	1723 reflections with *I* > 2σ(*I*)
Monochromator: Graphite	*R*_int_ = 0.030
Detector resolution: 7.31 pixels mm^-1^	θ_max_ = 25.2º
*T* = 296(2) K	θ_min_ = 2.3º
ω scans	*h* = −11→11
Absorption correction: multi-scan(Jacobson, 1998)	*k* = −9→8
*T*_min_ = 0.940, *T*_max_ = 0.959	*l* = −17→20
10679 measured reflections	

### Refinement

**Table:**

Refinement on *F*^2^	Secondary atom site location: Difmap
Least-squares matrix: Full	Hydrogen site location: Geom
*R*[*F*^2^ > 2σ(*F*^2^)] = 0.055	H-atom parameters constrained
*wR*(*F*^2^) = 0.174	*w* = 1/[σ^2^(*F*_o_^2^) + (0.1001*P*)^2^ + 0.236*P*] where *P* = (*F*_o_^2^ + 2*F*_c_^2^)/3
*S* = 1.08	(Δ/σ)_max_ = 0.001
2345 reflections	Δρ_max_ = 0.38 e Å^−^^3^
168 parameters	Δρ_min_ = −0.34 e Å^−^^3^
48 restraints	Extinction correction: none
Primary atom site location: Direct	

### Special details

**Table:**

Geometry. All s.u.'s (except the s.u. in the dihedral angle between two l.s. planes) are estimated using the full covariance matrix. The cell s.u.'s are taken into account individually in the estimation of s.u.'s in distances, angles and torsion angles; correlations between s.u.'s in cell parameters are only used when they are defined by crystal symmetry. An approximate (isotropic) treatment of cell s.u.'s is used for estimating s.u.'s involving l.s. planes.
Refinement. Refinement of *F*^2^ against ALL reflections. The weighted *R*–factor *wR* and goodness of fit *S* are based on *F*^2^, conventional *R*–factors *R* are based on *F*, with *F* set to zero for negative *F*^2^. The threshold expression of *F*^2^ > σ(*F*^2^) is used only for calculating *R*–factors(gt) *etc*. and is not relevant to the choice of reflections for refinement. *R*–factors based on *F*^2^ are statistically about twice as large as those based on *F*, and *R*–factors based on ALL data will be even larger.

### Fractional atomic coordinates and isotropic or equivalent isotropic displacement parameters (Å^2^)

**Table:**

	*x*	*y*	*z*	*U*_iso_*/*U*_eq_	Occ. (<1)
C1	0.0039 (3)	0.8100 (3)	0.27207 (16)	0.0699 (7)	
C2	−0.1128 (4)	0.9141 (5)	0.2543 (2)	0.0945 (10)	
H2	−0.1080	0.9916	0.2152	0.113*	
C3	−0.2344 (5)	0.9061 (6)	0.2925 (3)	0.1194 (14)	
H3	−0.3115	0.9773	0.2789	0.143*	
C4	−0.2447 (5)	0.7966 (7)	0.3498 (3)	0.1225 (15)	
H4	−0.3278	0.7927	0.3761	0.147*	
C5	−0.1305 (5)	0.6901 (6)	0.3689 (2)	0.1170 (14)	
H5	−0.1370	0.6138	0.4084	0.140*	
C6	−0.0049 (4)	0.6957 (4)	0.3296 (2)	0.0888 (10)	
H6	0.0716	0.6229	0.3423	0.107*	
C7	0.1365 (3)	0.8216 (3)	0.22835 (17)	0.0700 (7)	
H7	0.1517	0.9376	0.2146	0.084*	
C8	−0.0806 (5)	0.7118 (5)	0.0237 (2)	0.1202 (15)	
H8A	−0.0072	0.6701	−0.0078	0.144*	
H8B	−0.1403	0.6191	0.0370	0.144*	
C9	−0.1684 (5)	0.8298 (5)	−0.0188 (2)	0.1294 (16)	
H9A	−0.2427	0.8689	0.0118	0.194*	
H9B	−0.2128	0.7791	−0.0648	0.194*	
H9C	−0.1093	0.9213	−0.0322	0.194*	
C10	0.3970 (12)	0.693 (3)	0.1245 (10)	0.128 (2)	0.273 (7)
H10A	0.3922	0.6073	0.1630	0.153*	0.273 (7)
H10B	0.4512	0.7864	0.1477	0.153*	0.273 (7)
C11	0.4683 (18)	0.629 (2)	0.0560 (10)	0.131 (2)	0.273 (7)
H11A	0.4753	0.7168	0.0194	0.197*	0.273 (7)
H11B	0.4110	0.5404	0.0325	0.197*	0.273 (7)
H11C	0.5638	0.5889	0.0725	0.197*	0.273 (7)
C10'	0.3723 (5)	0.6344 (7)	0.0883 (5)	0.122 (2)	0.727 (7)
H10C	0.3621	0.5770	0.0394	0.147*	0.727 (7)
H10D	0.3777	0.5529	0.1293	0.147*	0.727 (7)
C11'	0.5025 (6)	0.7386 (9)	0.0942 (5)	0.136 (2)	0.727 (7)
H11D	0.4976	0.8149	0.0518	0.204*	0.727 (7)
H11E	0.5872	0.6700	0.0928	0.204*	0.727 (7)
H11F	0.5081	0.7992	0.1416	0.204*	0.727 (7)
O1	0.2648 (2)	0.7606 (3)	0.26977 (14)	0.0876 (7)	
H1	0.3101	0.8380	0.2908	0.131*	
O2	0.1031 (2)	0.5201 (2)	0.15362 (11)	0.0787 (6)	
O3	−0.0104 (2)	0.7844 (3)	0.09335 (11)	0.0889 (7)	
O4	0.2499 (3)	0.7456 (3)	0.09497 (16)	0.1077 (8)	
P1	0.11894 (8)	0.69802 (9)	0.14126 (4)	0.0710 (3)	

### Atomic displacement parameters (Å^2^)

**Table:**

	*U*^11^	*U*^22^	*U*^33^	*U*^12^	*U*^13^	*U*^23^
C1	0.0760 (17)	0.0661 (16)	0.0641 (15)	0.0012 (13)	−0.0139 (13)	−0.0117 (12)
C2	0.097 (2)	0.101 (2)	0.084 (2)	0.0294 (19)	0.0000 (18)	−0.0047 (17)
C3	0.100 (3)	0.146 (4)	0.111 (3)	0.030 (3)	0.002 (2)	−0.027 (3)
C4	0.107 (3)	0.144 (4)	0.119 (3)	−0.012 (3)	0.027 (3)	−0.048 (3)
C5	0.143 (4)	0.119 (3)	0.091 (3)	−0.029 (3)	0.022 (3)	−0.005 (2)
C6	0.102 (2)	0.080 (2)	0.082 (2)	−0.0091 (17)	−0.0077 (19)	0.0029 (16)
C7	0.0726 (16)	0.0543 (14)	0.0788 (17)	0.0010 (12)	−0.0172 (14)	0.0031 (12)
C8	0.125 (3)	0.118 (3)	0.108 (3)	0.018 (2)	−0.044 (3)	−0.014 (2)
C9	0.148 (4)	0.129 (3)	0.100 (3)	0.009 (3)	−0.050 (3)	−0.001 (2)
C10	0.093 (3)	0.113 (5)	0.179 (6)	0.013 (4)	0.031 (4)	0.015 (4)
C11	0.098 (4)	0.117 (5)	0.181 (6)	0.016 (4)	0.031 (4)	0.012 (4)
C10'	0.091 (3)	0.107 (4)	0.173 (6)	0.014 (2)	0.038 (3)	0.016 (3)
C11'	0.101 (3)	0.121 (4)	0.188 (6)	0.007 (3)	0.023 (3)	0.012 (4)
O1	0.0732 (12)	0.0743 (12)	0.1085 (16)	0.0046 (10)	−0.0301 (12)	−0.0079 (12)
O2	0.0826 (13)	0.0656 (12)	0.0845 (13)	0.0092 (9)	−0.0106 (10)	−0.0046 (9)
O3	0.1065 (16)	0.0916 (15)	0.0645 (11)	0.0306 (11)	−0.0157 (11)	−0.0027 (9)
O4	0.1062 (16)	0.0990 (16)	0.1221 (19)	0.0288 (14)	0.0325 (15)	0.0324 (15)
P1	0.0741 (5)	0.0677 (5)	0.0694 (5)	0.0125 (3)	−0.0035 (4)	0.0054 (3)

### Geometric parameters (Å, °)

**Table:**

C1—C6	1.371 (4)	C9—H9B	0.9600
C1—C2	1.380 (4)	C9—H9C	0.9600
C1—C7	1.503 (4)	C10—O4	1.471 (8)
C2—C3	1.358 (5)	C10—C11	1.509 (10)
C2—H2	0.9300	C10—H10A	0.9700
C3—C4	1.344 (7)	C10—H10B	0.9700
C3—H3	0.9300	C11—H11A	0.9600
C4—C5	1.378 (7)	C11—H11B	0.9600
C4—H4	0.9300	C11—H11C	0.9600
C5—C6	1.401 (5)	C10'—O4	1.456 (5)
C5—H5	0.9300	C10'—C11'	1.464 (7)
C6—H6	0.9300	C10'—H10C	0.9700
C7—O1	1.420 (3)	C10'—H10D	0.9700
C7—P1	1.814 (3)	C11'—H11D	0.9600
C7—H7	0.9800	C11'—H11E	0.9600
C8—C9	1.418 (5)	C11'—H11F	0.9600
C8—O3	1.450 (4)	O1—H1	0.82
C8—H8A	0.9700	O2—P1	1.462 (2)
C8—H8B	0.9700	O3—P1	1.561 (2)
C9—H9A	0.9600	O4—P1	1.562 (3)
			
C6—C1—C2	118.6 (3)	H9A—C9—H9B	109.5
C6—C1—C7	121.2 (3)	C8—C9—H9C	109.5
C2—C1—C7	120.2 (3)	H9A—C9—H9C	109.5
C3—C2—C1	121.6 (4)	H9B—C9—H9C	109.5
C3—C2—H2	119.2	O4—C10—C11	105.9 (11)
C1—C2—H2	119.2	O4—C10—H10A	110.5
C4—C3—C2	120.9 (4)	C11—C10—H10A	110.5
C4—C3—H3	119.6	O4—C10—H10B	110.5
C2—C3—H3	119.6	C11—C10—H10B	110.5
C3—C4—C5	119.1 (4)	H10A—C10—H10B	108.7
C3—C4—H4	120.4	O4—C10'—C11'	106.2 (5)
C5—C4—H4	120.4	O4—C10'—H10C	110.5
C4—C5—C6	120.7 (4)	C11'—C10'—H10C	110.5
C4—C5—H5	119.6	O4—C10'—H10D	110.5
C6—C5—H5	119.6	C11'—C10'—H10D	110.5
C1—C6—C5	119.1 (4)	H10C—C10'—H10D	108.7
C1—C6—H6	120.5	C10'—C11'—H11D	109.5
C5—C6—H6	120.5	C10'—C11'—H11E	109.5
O1—C7—C1	113.6 (2)	H11D—C11'—H11E	109.5
O1—C7—P1	104.17 (19)	C10'—C11'—H11F	109.5
C1—C7—P1	112.03 (18)	H11D—C11'—H11F	109.5
O1—C7—H7	109.0	H11E—C11'—H11F	109.5
C1—C7—H7	109.0	C7—O1—H1	109.5
P1—C7—H7	109.0	C8—O3—P1	122.2 (2)
C9—C8—O3	111.1 (3)	C10'—O4—P1	122.1 (3)
C9—C8—H8A	109.4	C10—O4—P1	118.8 (8)
O3—C8—H8A	109.4	O2—P1—O3	115.86 (12)
C9—C8—H8B	109.4	O2—P1—O4	114.24 (13)
O3—C8—H8B	109.4	O3—P1—O4	101.79 (14)
H8A—C8—H8B	108.0	O2—P1—C7	114.79 (12)
C8—C9—H9A	109.5	O3—P1—C7	102.18 (12)
C8—C9—H9B	109.5	O4—P1—C7	106.42 (15)

### Hydrogen-bond geometry (Å, °)

**Table:**

*D*—H···*A*	*D*—H	H···*A*	*D*···*A*	*D*—H···*A*
O1—H1···O2^i^	0.82	1.90	2.716 (3)	174

Symmetry codes: (i) −*x*+1/2, *y*+1/2, −*z*+1/2.
